# FOXC2 Promotes Vasculogenic Mimicry in Ovarian Cancer

**DOI:** 10.3390/cancers14194851

**Published:** 2022-10-04

**Authors:** Maria Sol Recouvreux, Jiangyong Miao, Maricel C. Gozo, Jingni Wu, Ann E. Walts, Beth Y. Karlan, Sandra Orsulic

**Affiliations:** 1 Department of Obstetrics and Gynecology, David Geffen School of Medicine, University of California Los Angeles, Los Angeles, CA 90095, USA; 2 Department of Pathology, Massachusetts General Hospital, Boston, MA 02114, USA; 3 Women’s Cancer Program, Cedars-Sinai Medical Center, Los Angeles, CA 90048, USA; 4 Department of Pathology and Laboratory Medicine, Cedars-Sinai Medical Center, Los Angeles, CA 90048, USA; 5 Jonsson Comprehensive Cancer Center, University of California Los Angeles, Los Angeles, CA 90095, USA; 6 Veterans Administration Greater Los Angeles Healthcare System, Los Angeles, CA 90095, USA

**Keywords:** angiogenesis, vasculogenesis, vascular, epithelial-mesenchymal transition, ovarian cancer progression, stem cells, mesenchymal cells, vasculogenic mimicry, FOXC2

## Abstract

**Simple Summary:**

Tumors need a continuous supply of oxygen and nutrients to sustain growth. One coping mechanism is to secret factors that promote the development of new blood vessels. However, this may not be sufficient for the growth of highly aggressive tumors. Vasculogenic mimicry is another coping mechanism whereby cancer cells form vascular-like structures capable of carrying blood and nutrients. Expression of nuclear FOXC2 has been associated with aggressiveness and advanced stage in most cancers, including ovarian cancer. We confirmed most of the known mechanisms by which FOXC2 promotes cancer aggressiveness. Additionally, we found evidence that FOXC2 expression is associated with vasculogenic mimicry in ovarian cancer samples and that FOXC2 overexpression promotes vasculogenic mimicry in cell culture.

**Abstract:**

FOXC2 is a forkhead family transcription factor that plays a critical role in specifying mesenchymal cell fate during embryogenesis. FOXC2 expression is associated with increased metastasis and poor survival in various solid malignancies. Using in vitro and in vivo assays in mouse ovarian cancer cell lines, we confirmed the previously reported mechanisms by which FOXC2 could promote cancer growth, metastasis, and drug resistance, including epithelial-mesenchymal transition, stem cell-like differentiation, and resistance to anoikis. In addition, we showed that FOXC2 expression is associated with vasculogenic mimicry in mouse and human ovarian cancers. FOXC2 overexpression increased the ability of human ovarian cancer cells to form vascular-like structures in vitro, while inhibition of FOXC2 had the opposite effect. Thus, we present a novel mechanism by which FOXC2 might contribute to cancer aggressiveness and poor patient survival.

## 1. Introduction

FOXC2 is a transcription factor within the large family of forkhead box (FOX) genes. During embryogenesis, FOXC2 is expressed in the developing mesenchyme [1] and is required for aortic arch formation, blood and lymphatic vessel remodeling, arterial-venous specification, and skeletogenesis [2,3,4,5]. Through its role in fat and glucose metabolism, FOXC2 is also an important arbiter of energy storage and expenditure in adult tissues. FOXC2 inhibits adipogenic differentiation in vitro [6,7] and transgenic mice with adipocyte-specific FOXC2 overexpression exhibit a lean phenotype and are protected against diet-induced insulin resistance [7,8,9]. Sustained FOXC2 expression suppresses myogenesis in myoblast cells and alters their lineage commitment toward osteogenesis by inducing the Wnt4 and Bmp4 signaling pathways [10]. Given the multiple functions of FOXC2, it is not surprising that its functional loss is associated with a wide array of human conditions and diseases. For example, a frameshift mutation of FOXC2 is associated with lymphedema-distichiasis and diabetes [11] while the FOXC2 C-512T polymorphism is associated with obesity, dyslipidemia, and diabetes [12,13,14]. Cancer is known to reactivate many pathways that are important in embryonic development. Elevated expression of FOXC2 mRNA and protein correlate with tumor aggressiveness, metastasis, and poor prognosis in many cancer types, including breast cancer [15,16], prostate cancer [17], pancreatic cancer [18], colon cancer [19], gastric cancer [20], non-small-cell lung cancer [21,22], ovarian cancer [23,24], esophageal squamous cell carcinoma [25], osteosarcoma [26], glioma [27], glioblastoma [28], melanoma [29], hepatocellular carcinoma [30], and extrahepatic cholangiocarcinoma [31]. FOXC2 is known to aid in tumor progression by promoting epithelial-mesenchymal transition (EMT) [19,21,22,32,33], stem cell differentiation [26,34], proliferation and migration [18,28,30], drug resistance [20,23,24], and resistance to anoikis, a form of programmed cell death that occurs when cells detach from the extracellular matrix [26].

Analogous to its role in embryonal angiogenesis and lymphangiogenesis, FOXC2 plays a critical role in promoting the growth of new vessels thereby establishing the obligatory blood supply for tumor progression [3,4,5,20,35,36,37,38,39,40,41]. Although anti-angiogenic therapies, such as anti-VEGF antibodies, are prescribed to inhibit neovascularization and decrease the blood supply needed for growth in various types of cancer, the results have been disappointing in highly invasive and aggressive cancers, including ovarian cancer. While anti-VEGF therapy improves progression-free survival and quality of life in ovarian cancer patients, it does not improve overall survival [42]. Tumor regrowth in the presence of angiogenesis inhibitors suggests that tumor cells can acquire alternate ways to obtain an adequate blood supply. Indeed, anti-angiogenic therapies specifically target endothelial cells, whereas cancer cells can transdifferentiate and form de novo functional vascular channels independent of endothelial cells, a phenomenon known as vasculogenic mimicry (VM) [43]. It has been shown that short-term treatment of mice with bevacizumab (anti-VEGF therapy) increased metastasis and induced hypoxia and VM, suggesting that VM could be a key alternative mechanism underlying tumor resistance to anti-angiogenic therapy [44]. VM has been observed in multiple types of highly aggressive solid cancers and is typically associated with treatment resistance [45] and poor patient prognosis [46]. VM has been reported in 25–37% of ovarian cancers and is correlated with hypoxia, EMT, and poor patient prognosis [47,48,49,50,51]. Inhibition of VM in ovarian cancer is an attractive potential therapeutic strategy to overcome the limitations of anti-angiogenic therapies and eradicate blood and nutrient supplies to vulnerable tumors [52]. However, clinical trials targeting VM in ovarian cancer are currently hampered by a limited understanding of molecular mechanisms and associated signaling pathways that regulate VM. Cancer cells involved in VM have been shown to express markers associated with EMT and cancer stem cells (CSCs) [43]. Here, we show that the EMT and CSC marker, FOXC2, is involved in VM in ovarian cancer.

## 2. Materials and Methods

### 2.1. Xenograft Experiments in Mice

All animal procedures were performed in accordance with the NIH Guide for the Care and Use of Laboratory Animals and approved by the Massachusetts General Hospital Subcommittee on Research Animals (SRAC). Tumor cells were harvested by brief incubation with 0.05% trypsin with 0.53 mM EDTA (Cellgro, Lincoln, NE, USA), washed in phosphate-buffered saline (PBS), and resuspended in PBS (Gibco/Thermo Fisher, Waltmam, MA, USA). 5 × 10^6^ cells in a 0.2 mL volume were injected subcutaneously or intraperitoneally into the hind flanks of 6-week-old female Nu/Nu mice (Charles River Laboratories, Wilmington, MA, USA). The mice were monitored for tumor formation and the accumulation of ascites. Subcutaneous tumor volume was determined using the equation V = (W(2) × L)/2. At the end of the experiment, the mice were euthanized by CO_2_ inhalation followed by cervical dislocation and the excised tumors were fixed in 1:10 buffered formalin at 4 °C overnight, then transferred to 70% ethanol before embedding into paraffin blocks.

### 2.2. Immunohistochemical Staining of Human Specimens and Immunofluorescence Staining of Cells in Culture

Formalin-fixed paraffin-embedded (FFPE) slides were deparaffinized in a graded xylene/ethanol series and the epitopes were unmasked by microwaving the slides in citrate buffer (pH 6.0) (Vector Laboratories, Burlingame, CA, USA). Endogenous peroxidase activity was blocked with 3% hydrogen peroxide after which the slides were incubated for 20 min in 5% dry milk in PBS to reduce nonspecific background staining. The human-specific mouse monoclonal FOXC2 antibody obtained from Dr. Naoyuki Miura was used at a 1:100 dilution and incubated at room temperature for 30 min. Primary antibodies were detected by an anti-mouse horseradish peroxidase-labeled secondary antibody (Vector Laboratories) for 1 h. The slides were stained for 5 min with 0.05% 3′,3-diaminobenzidine tetrahydrochloride (DAB HCl, Vector Laboratories), then counterstained with hematoxylin, dehydrated, and mounted with coverslips. Immunofluorescence microscopy with anti-E-cadherin (BD Transduction Laboratories, Franklin Lakes, NJ, USA) was done as described in our previous publication [53].

### 2.3. Periodic Acid Schiff (PAS) and CD31 Double Staining for VM Detection

PAS and CD31 detection in FFPE slides was conducted as previously described [54,55]. The slides were first immunostained with CD31 (Abcam, Cambridge, UK, ab124432) as described above. After secondary antibody incubation, PAS staining was performed following the manufacturer’s recommendations (Sigma, St. Louis, MO, USA, 395B-1KT). Briefly, the slides were incubated for 5 min at room temperature with PAS, washed, and incubated for 15 min with Schiff’s Reagent. The slides were then counterstained with hematoxylin, dehydrated, and mounted.

### 2.4. Cell Lines

Generation of the mouse ovarian cancer cell lines C1, C11, C111, C2, C22, T1, T11, T2, BR2, and BR5 has been described in our previous publications [56,57,58,59]. The SK-OV-3 (SKOV3) and TYK-nu (TYKNU) cell lines were purchased from the American Type Culture Collection (ATCC, Manassas, VA) and Fisher Scientific, respectively. Upon receipt, the cell lines were expanded into multiple frozen vials. A fresh vial was used for each experiment. All mouse cell lines were cultured in Dulbecco’s Modified Eagle Medium (DMEM, Gibco) with 10% fetal bovine serum (FBS, Gibco) and 1% penicillin-streptomycin (Gibco) unless otherwise indicated.

### 2.5. Retroviral and Lentiviral Infection

Detailed methods for retroviral and lentiviral infection have been described in our previous publications [10,26]. The retroviral vectors pWZL-blast and pWZL-FOXC2 were provided by Dr. Sendurai Mani. The lentiviral plasmids pLKO.1 scrambled and pLKO.1-FOXC2 sh were obtained from Sigma.

### 2.6. Cell Proliferation

To monitor cell proliferation, cells were plated in triplicate at 4 × 10^4^ cells per cm^2^ and harvested every day. Fresh media was added daily. For quantification of short-term cell proliferation (72 h) in 96-well plates, CellTiter-Glo luminescent cell viability assay (Promega, Madison, WI, USA) was used following the manufacturer’s recommendations. For long-term cell proliferation (7 days), cells harvested from 6-well plates were stained with trypan blue and the number of live cells was counted using hematocytometer.

### 2.7. Soft Agar Assay

Cell growth in soft agar was assessed by counting the number of colonies formed in 0.5% agarose with a 0.09% agarose base layer as described in our previous publication [26]. Cells were mixed with agarose at a density of 1 × 10^4^ cells per well and plated in triplicate in 6-well plates. Plates were incubated for 3 weeks at 37 °C in a 5% CO_2_ incubator. Fresh media was added every 2–5 days. Colonies were counted using a stereomicroscope.

### 2.8. Anchorage-Independent Growth Assay

1 × 10^4^ cells per well were seeded in ultra-low attachment 6-well plates (Corning) and incubated with 3 mL DMEM at 37 °C in a 5% CO_2_ incubator. The growth media were changed daily. After 5 days, the media were collected in 15 mL conical tubes, and the cells were precipitated for 0.5 min at 1000 rpm. Live cells were quantified using trypan blue and propidium iodide (PI) staining.

### 2.9. Wound-Healing Scratch Assay

Cells were seeded at 6 × 10^4^ cells per well in a 12-well plate. After reaching confluence, the plate was scratched manually with a pipette tip and the media was replaced before photographing the plates under the microscope. To minimize detachment of FOXC2-expressing cells from the plates, the cells were kept undisturbed for two days after which they were taken out of the incubator and photographed.

### 2.10. Vasculogenic Tube Formation Assay

SKOV3 and TYKNU cells were seeded at 7 × 10^4^ and 5 × 10^4^ cells/well, respectively, in 96-well plates coated with 50 µL of Matrigel that was polymerized at 37 °C for 30 min as previously described [60]. Cells were incubated for 24 to 48 h in a MuviCyte (PerkinElmer, Waltham, MA, USA) incubator at 37 °C in 5% CO_2_. Microphotographs were taken every 3 h by the MuviCyte live cell imaging software (PerkinElmer) and analyzed in Image J with an angiogenesis plugin, as previously described [61].

### 2.11. Western Blot Analysis

Cell lysates were prepared using RIPA lysis buffer (Sigma) containing a protease/phosphatase inhibitor cocktail (Roche, Basel, Switzerland). Protein concentrations were quantified using the BCA protein assay (Pierce, Appleton, WI, USA). For each cell line, 10 µg of lysate was run in a 4–20% polyacrilamide gradient gel (Bio-Rad, Hercules, CA, USA) and transferred into a nitrocellulose membrane (Bio-Rad). The membrane was blocked in 5% milk in PBS with 0.1% Tween 20 (PBS-T) for 1 h and incubated with the appropriate antibody overnight at 4 °C. FOXC2 (obtained from Dr. Naoyuki Miura) and α-tubulin (Sigma) were used at a 1:1000 dilution. Membranes were washed with PBS-T and incubated with secondary antibodies for 1 h at room temperature, after which they were washed again with PBS-T. The Western blots were scanned and analyzed using an enhanced chemiluminescence (ECL) detection system (Amersham, Amersham, UK) following the protocol provided by the manufacturer. α-tubulin was used as an internal control for protein loading and normalization.

### 2.12. Quantitative Real-Time Polymerase Chain Reaction (qRT-PCR)

RNA was extracted from cells using the RNeasy Kit (Qiagen, Hilden, Germany) and reverse transcribed to cDNA using the Quantitect Reverse Transcription Kit (Qiagen). For qRT-PCR, 50 ng of cDNA was mixed with the appropriate primers in a 96-well plate containing iQ SYBR-Green Supermix (BioRad, Hercules, CA, USA). The qRT-PCR reaction was performed using an iCycler optical module and thermocycler (BioRad). Expression levels of FOXC2 (forward: GCCTAAGGACCTGGTGAAGC, reverse: TTGACGAAGCACTCGTTGAG) were normalized to GAPDH or RPL32 as described in our previous publications [10,26].

### 2.13. RNA Sequencing (RNA-Seq) and Differential Gene Expression Analysis

Libraries for RNA-Seq were prepared with the Illumina TruSeq Stranded mRNA LP Kit (20020595). After mRNA enrichment and fragmentation, first-strand cDNA was synthesized using random priming. dUTP was incorporated into the second cDNA strand by second-strand synthesis. This was followed by the generation of blunt ends through end repair, A-tailing, adaptor ligation, and PCR amplification. Single-lane multiplexing of samples was achieved by using different adaptors in one lane. Sequencing was performed on Illumina HiSeq 3000 for SE 1 × 50 run. Data quality was verified using Illumina SAV. Demultiplexing was conducted with Illumina Bcl2fastq v2.19.1.403 software. The reads were mapped using STAR 2.27a and Ensembl GRCh38.98 GTF file was used to quantify read counts per gene. Partek flow software version 7.0 was used to determine read counts normalized by CPM + 1.0 × 10^−4^. The differential gene expression analysis between two groups of samples (10 GFP-expressing and 10 FOXC2-expressing cell lines) was conducted using the Limma test in R2 (https://hgserver2.amc.nl/cgi-bin/r2/main.cgi, accessed on 1 May 2022). False Discovery Rate (FDR) was used for multiple testing correction.

### 2.14. Statistical Analysis

Data were expressed as means ± SEM of three or more independent experiments unless otherwise stated. Statistical analyses were performed using GraphPad Prism (version 9.0; GraphPad Software). Statistically significant data were assessed by unpaired Student’s *t*-test unless otherwise noted. Intergroup differences were considered statistically significant at *p* ≤ 0.05.

## 3. Results

### 3.1. Ectopic Expression of FOXC2 in Mouse Ovarian Cancer Cells Alters Cell Morphology and Increases Cell Proliferation, Anchorage-Independent Growth, and Resistance to Anoikis

**Figure 1:**
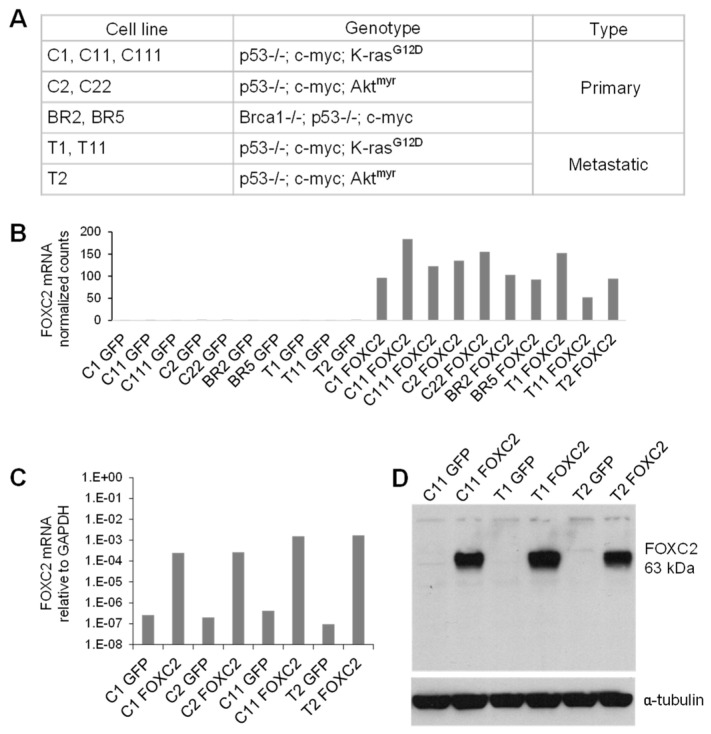
FOXC2 overexpression in genetically defined mouse ovarian cancer cell lines. (**A**) A table of genetically defined mouse ovarian cancer cell lines used in this study. (**B**) RNA-seq detection of FOXC2 in mouse ovarian cancer cell lines after transduction with GFP or FOXC2. (**C**) Quantitative RT-PCR (average of two replicates normalized to GAPDH) and (**D**) Western blot detection of FOXC2 in selected mouse ovarian cancer cell lines expressing GFP or FOXC2. α-tubulin was used as a loading control. The uncropped blots are shown in File S1.

To investigate the potential function(s) of FOXC2 in ovarian cancer and the possible synergism or reliance on genes that are frequently altered in ovarian cancers, such as myc, Akt, Kras, and Brca1, ten genetically defined primary and metastatic mouse ovarian cancer cell lines [56,57] were retrovirally transduced with human FOXC2 or green fluorescent protein (GFP) control (Figure 1A). The expression of FOXC2 was confirmed by RNA sequencing (Figure 1B). Two to five cell lines were randomly selected for subsequent analyses, including qRT-PCR (Figure 1C) and Western blot (Figure 1D) validation of FOXC2 expression.

**Figure 2:**
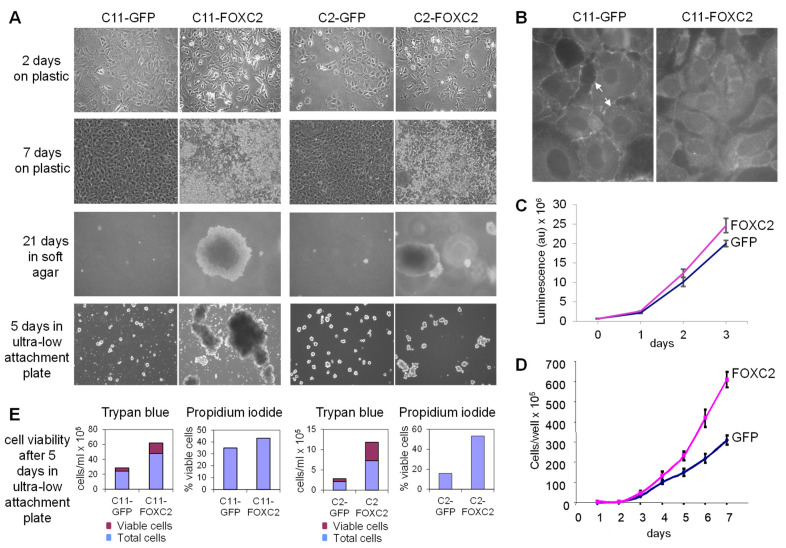
FOXC2 overexpression in mouse ovarian cancer cells alters cell morphology and facilitates anchorage-independent growth in vitro. (**A**) Photomicrographs of C11 and C2 ovarian cancer cell lines with ectopic expression of GFP or FOXC2 in different growth conditions. (**B**) Immunofluorescence detection of E-cadherin in sub-confluent cell culture. The arrows indicate E-cadherin localization in membranes with and without cell–cell interaction. (**C**) Cell proliferation within the first three days after plating the cells on plastic. The growth curves were generated using the CellTiterGlo kit and measuring the luminescent signal. (**D**) Cell proliferation within seven days after plating the cells on plastic. The growth curves were generated by manual counting of viable cells after harvesting and staining with trypan blue. (**E**) The viability of cells grown for five days in ultra-low attachment plates. Assays were performed using trypan blue and propidium iodide (PI).

To determine whether FOXC2 expression in our model system has similar effects to those previously observed in various human cancer cell lines, we evaluated the cells for changes in morphology and growth patterns on plastic, soft agar, and ultra-low attachment plates. After two days of growth on plastic, GFP-expressing cell lines exhibited a cobblestone-like epithelial morphology while FOXC2-expressing cell lines exhibited a spindle-like morphology and reduced cell–cell contact (Figure 2A). C11-GFP cells expressed membrane E-cadherin while C11-FOXC2 cells only exhibited cytoplasmic E-cadherin, characteristic of EMT (Figure 2B). Upon reaching confluence on day three after plating, C11-FOXC2 cells started exhibiting proliferative advantage over C11-GFP cells (Figure 2C,D). After seven days of growth on plastic, GFP-expressing cells continued to proliferate as a confluent monolayer while FOXC2-expressing cells accelerated their proliferation rate (Figure 2D) and formed three-dimensional aggregates (Figure 2A). In a wound-healing scratch assay, confluent C11-GFP cells migrated to the clear area of the plate while C11-FOXC2 cells detached from the confluent layer and randomly re-seeded the plate (Figure S1). FOXC2-expressing cells grew colonies in soft agar (Figure 2A) and formed viable aggregates in ultra-low attachment plates (Figure 2A,E). There was no appreciable difference in morphology or growth patterns based on specific genetic alterations and primary/metastatic status. Together, these data show that the FOXC2-expressing mouse ovarian cancer cell lines exhibit phenotypic characteristics similar to those described in human ovarian, breast, and other cancer cell lines [62].

### 3.2. Ectopic Expression of FOXC2 in Mouse Ovarian Cancer Cells Enhances Tumor Growth

**Figure 3:**
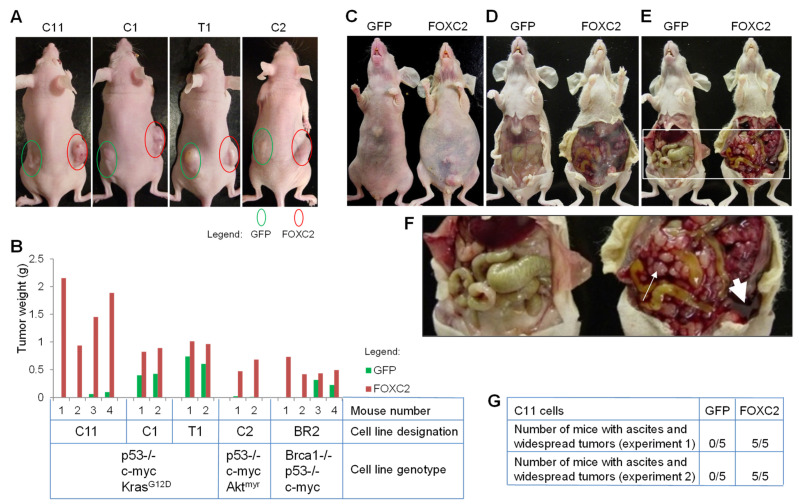
Ectopic FOXC2 expression facilitates tumor growth in vivo. (**A**) Representative images of tumors in four different mouse ovarian cancer cell lines 18 days after subcutaneous injection of 5 × 10^6^ cells expressing GFP (left flank) or FOXC2 (right flank). (**B**) Wet tumor weight 18 days after subcutaneous injection of the indicated ovarian cancer cell lines. (**C**) Representative image of mice 20 days after intraperitoneal injection of 5 × 10^6^ C11-GFP and C11-FOXC2 cell lines. Abdominal bloating is visible in the mouse injected with C11-FOXC2 cells. (**D**) Image of the same mice after removing the abdominal skin. The presence of hemorrhagic ascites is apparent in the abdomen of the mouse injected with C11-FOXC2 cells. (**E**) Image of the same mice after opening the peritoneal cavity. (**F**) Enlarged image of the area labeled with a white square in (**E**). Tumors in mice injected with C11-GFP cells are microscopic and confined to the omentum while the abdomen of mice injected with C11-FOXC2 cells is packed with numerous small tumor nodules (thin arrow) and hemorrhagic ascites (thick arrow). (**G**) Quantification of mice with ascites and widespread intraperitoneal tumors 20 days after intraperitoneal injection of cancer cells.

To gain further insight into the potential role of FOXC2 in ovarian cancer progression, we analyzed the effects of FOXC2 overexpression on tumor formation in xenografts in nude mice. Eighteen days after subcutaneous injection of cancer cells, the FOXC2-expressing cells had formed significantly larger tumors than the GFP-expressing cells (Figure 3A). The more rapid formation of subcutaneous tumors in the presence of FOXC2 was confirmed in four additional primary mouse ovarian cancer cell lines that contained different combinations of genetic alterations (Figure 3B). Twenty days after intraperitoneal injection into nude mice, C11-GFP cells had formed tumors in the omentum while mice injected with C11-FOXC2 cells had developed extensive ascites and macroscopic tumor nodules throughout the peritoneal cavity (Figure 3C–G).

### 3.3. Ectopic Expression of FOXC2 in Mouse Ovarian Cancer Cells Induces Expression of EMT, CSC, and Angiogenesis-Related Genes

**Figure 4:**
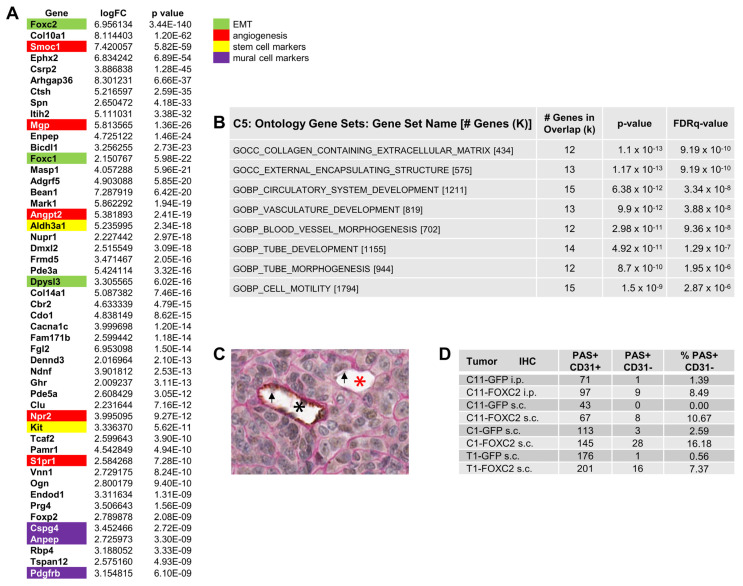
FOXC2-expressing mouse ovarian cancer cell lines have increased expression of angiogenesis-related markers and exhibit VM in mouse xenografts. (**A**) Differentially expressed genes between genetically defined mouse ovarian cancer cell lines transduced with GFP (n = 10) and FOXC2 (n = 10). (**B**) Ontology Gene Sets (C5) analysis of the upregulated genes shown in (**A**). (**C**) PAS (pink) and CD31 (brown) double staining of a subcutaneous C11-FOXC2 mouse tumor. The PAS+/CD31+ tubular structure (black asterisk) indicates a conventional blood vessel lined by CD31-positive endothelial cells. The adjacent PAS+/CD31- tubular structure (red asterisk) is indicative of VM. (**D**) PAS and CD31 scores table. In each tumor, all recognizable PAS+ tubular structures with or without erythrocytes in the lumen were assessed for CD31 staining. The difference in the percent of PAS+/CD31- tubular structures between GFP- and FOXC2-expressing tumors is significant, *p* = 0.003. The arrows indicate erythrocytes.

To identify pathways altered by FOXC2 expression, the ten genetically defined mouse ovarian cancer cell lines shown in Figure 1A transduced with FOXC2 or GFP were analyzed by RNA-seq (Table S1). The gene expression changes observed in association with FOXC2 expression appeared to be independent of the preexisting genetic alterations and primary/metastatic status of the cell lines (Figure 1A and Table S1). Among the highly downregulated genes were epithelial and mesothelial cell markers, such as E-cadherin (Cdh1), Cytokeratin 8 and 18 (Krt8 and Krt18), Mesothelin (Msln), and CA125 (Muc16) (Table S1). Downregulation of these cell markers suggested that FOXC2 overexpression induced trans-differentiation of the mouse ovarian cancer cell lines. Genes upregulated in FOXC2-expressing cell lines included several CSC marker genes, such as aldehyde dehydrogenase 3 family member A1 (Aldh3a1) and KIT proto-oncogene receptor tyrosine kinase (Kit) as well as EMT-associated genes Foxc1 [63,64] and Dpysl3 [65,66] (Figure 4A). These results are consistent with a previous study reporting that FOXC2 overexpression in immortalized human mammary epithelial cells induced expression of CSC and EMT markers [67]. Genes expressed in lymphovascular cells and/or involved in angiogenesis, including SPARC related modular calcium binding-1 (Smoc1) [68,69], matrix GLA protein (Mgp) [70,71], angiopoietin 2 (Angpt2) [72,73], VEGFRs co-receptor neuropilin-2 (Nrp2) [74], and sphingosine-1-phosphate receptor 1 (S1pr1) [75] were also significantly upregulated in FOXC2-expressing ovarian cancer cells (Figure 4A). Additionally, mural cell markers [76], such as chondroitin sulfate proteoglycan 4 (Cspg4, also known as NG2), alanyl membrane aminopeptidase (Anpep, also known as CD13), and platelet-derived growth factor receptor beta (Pdgfrb), were upregulated in FOXC2-expressing ovarian cancer cells (Figure 4A). The Gene Ontology (GO) analysis of genes upregulated in FOXC2-expressing tumors indicated possible activation of pathways involved in blood vessel development and tube formation (Figure 4B). However, prototypical endothelial cell markers, such as platelet and endothelial cell adhesion molecule 1 (Pecam1, CD31), cluster of differentiation 34 (CD34), cadherin 5 (Cdh5, VE-cadherin), intercellular adhesion molecule 1 (Icam1), and vascular cell adhesion molecule 1 (Vcam1), were not altered by FOXC2 expression (Table S1). These results suggest that ectopic FOXC2 expression in mouse ovarian cancer cells affects molecular pathways associated with CSC, EMT, angiogenesis, and mural cell differentiation but does not appear to be associated with cancer-endothelial cell trans-differentiation.

### 3.4. FOXC2 Expression Is Associated with VM in Ovarian Cancer

Increased mRNA expression of the mural cell markers Cspg4 (NG2), Anpep (CD13), and Pdgfrb in FOXC2-expressing ovarian cancer cell lines (Figure 4A) suggested possible trans-differentiation of epithelial cancer cells into mural cells, a phenomenon that has been associated with VM [77]. Pdgfrb has been previously identified as a driver of VM in triple-negative breast cancer and glioblastoma through the trans-differentiation of cancer stem-like cells to mural cells [78,79]. Cspg4 has been associated with VM in angiotropic melanoma cells [80] and overexpression of Anpep in ovarian cancer cell lines has been associated with an enlarged vascular lumen in ovarian cancer xenografts [81]. To detect if FOXC2 expression is associated with the formation of VM structures in mouse tumors that were generated by intraperitoneal and subcutaneous injection of mouse ovarian cancer cell lines transduced with GFP or FOXC2, we double-stained tumor sections with CD31 antibody and PAS. Pathologic examination of tubular structures for PAS and CD31 staining (Figure 4C) showed that the percent of PAS-positive/CD31-negative tubular structures (indicative of VM) was significanly higher in FOXC2-expressing than in GFP-expressing tumors (Figure 4D).

**Figure 5:**
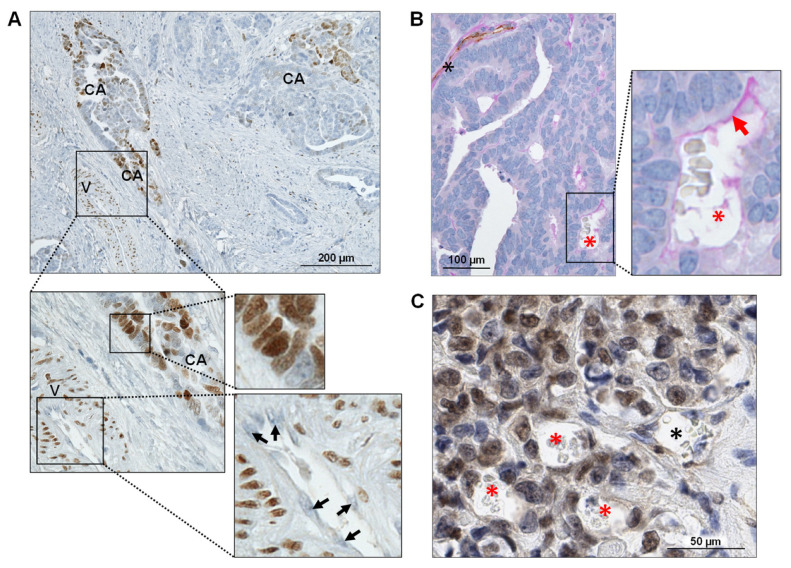
FOXC2 expression in human HGSOC is associated with VM. (**A**) Immunohistochemical staining of human ovarian cancer exhibiting nuclear FOXC2 expression in vascular smooth muscle cells (V) and a subset of carcinoma cells (CA). The panels show regions from the image at increased magnification. Arrows indicate endothelial cells, which do not express FOXC2. (**B**) PAS and CD31 double histochemical staining of FOXC2-expressing HGSOC. The black asterisk indicates a conventional PAS-positive/CD31-positive blood vessel. The red asterisk indicates an erythrocyte-containing tubular structure lined by CD31-negative/PAS-positive (red arrow) cancer cells indicative of VM. (**C**) Immunohistochemical staining of HGSOC exhibiting nuclear FOXC2 expression in cancer cells and VM. The black asterisk indicates a tubular structure containing erythrocytes and lined by endothelial cells while the red asterisks indicate erythrocyte-containing tubular structures that are lined by FOXC2-positive cancer cells.

To assess the frequency and spatial pattern of FOXC2 expression in the most common subtype of ovarian cancer, high-grade serous ovarian carcinoma (HGSOC), immunohistochemical analysis was performed on 50 HGSOC samples of which 20 were whole sections and 30 were arranged into a tissue microarray (TMA) (1.5 mm cores; no overlap with the whole-section samples). Consistent with the previously described role of FOXC2 in vascular development [4], nuclear FOXC2 staining was detected in vascular smooth muscle cells of most large blood vessels (V in Figure 5A) while endothelial cells did not express FOXC2 (arrows in Figure 5A). In addition, focal nuclear FOXC2 staining was observed in a subset of cancer cells in six of 50 samples (three of 20 whole-section samples and three of 30 TMA samples) (CA in Figure 5A). To detect if FOXC2 expression is associated with the formation of VM structures in human ovarian cancer, the TMA with 30 HGSOC samples was PAS/CD31 double stained. Using the criterion described above to identify VM, we observed VM in three of 30 HGSOC samples (Figure 5B). Two of these three samples exhibited focal nuclear FOXC2 staining in cancer cells and contained tubular structures with intraluminal red blood cells lined with FOXC2-expressing cancer cells (Figure 5C), which is indicative of VM.

### 3.5. Manipulation of FOXC2 Expression Levels in Human Ovarian Cancer Cell Lines Alters Their Vasculogenic Activity In Vitro

**Figure 6:**
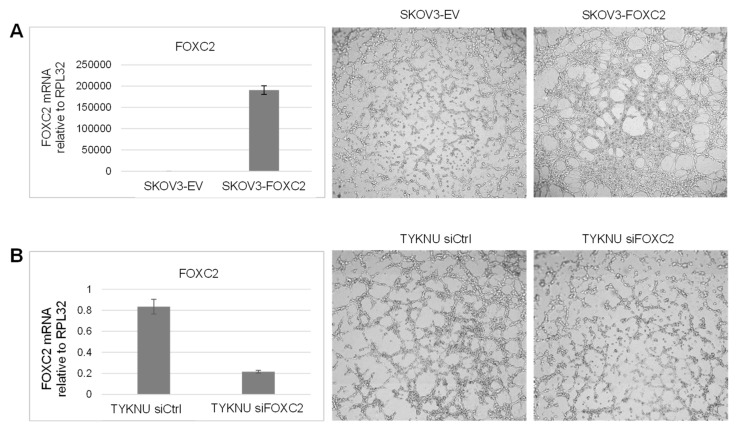
FOXC2 induces VM in human ovarian cancer cell lines. (**A**) Overexpression of FOXC2 in SKOV3 cells: qRT-PCR analysis and a representative image of a tube formation assay 6 h after plating an equal number (7 × 10^4^ cells/well in a 96-well plate) of SKOV3 cells transduced with an empty vector (EV) or FOXC2. (**B**) siRNA-mediated silencing of endogenous FOXC2 in TYKNU cells: qRT-PCR analysis and a representative image of a tube formation assay 6 h after plating an equal number (5x10^4^ cells/well in a 96-well plate) of TYKNU cells transduced with control siRNA (siC) or siRNA targeting FOXC2 (si FOXC2). The RNA levels were normalized to RPL32. Error bars represent standard deviation.

The vasculogenic activity of tumor cells can be assessed using the vasculogenic tube formation assay [82]. SKOV3 ovarian cancer cell lines have been previously shown to form vascular-like channels in this assay [83]. Overexpression of FOXC2 in SKOV3 cells did not affect proliferation within the first two days (Figure S2) but it did enhance the formation of tubular structures, which was apparent six hours after cell plating (Figure 6A) suggesting that FOXC2 promotes VM. To validate this result, we used the TYKNU cell line which, among HGSOC cell lines in the CCLE database [84], exhibited the highest levels of FOXC2 expression. We observed that TYKNU cells are capable of forming vascular-like structures in the vasculogenic tube formation assay and that siRNA-mediated silencing of endogenously expressed FOXC2 reduced the formation of tubular structures by TYKNU cells (Figure 6B). To quantify the vasculogenic activity, we measured mean mesh size, total length, total mesh area, total segment length, and the number of junctions in the tubular structures. Although differences in these parameters were not statistically significant, they showed a consistent trend with all vasculogenic activity parameters increased upon FOXC2 overexpression and decreased upon FOXC2 silencing (Figure S3).

## 4. Discussion

**Figure 7:**
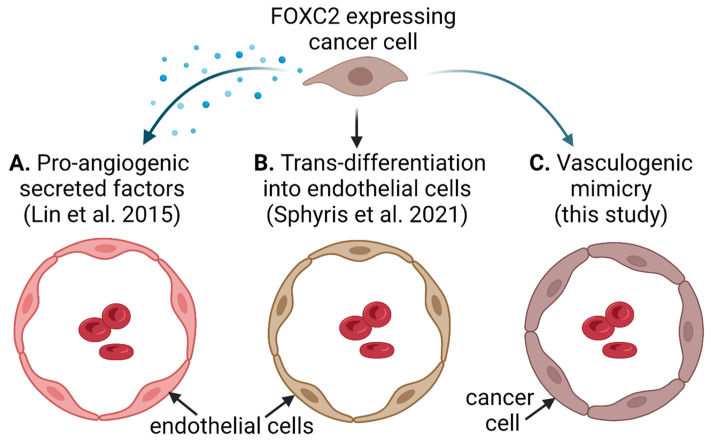
Three pathways by which FOXC2-expressing cancer cells promote tumor vascularization. (**A**) Cancer cells secrete factors that promote the growth of endothelial cells in a paracrine manner. (**B**) Cancer cells trans-differentiate into endothelial cells. (**C**) Cancer cells form endothelium-independent vascular channels. Created with BioRender.com [88,92].

FOXC2 has been proposed as a potential therapeutic target because it is involved in multiple processes that support cancer growth, metastasis, and chemotherapy resistance, such as EMT, CSC differentiation, angiogenesis, and resistance to anoikis [36,62,85,86]. Consistent with a previously identified association between FOXC2-induced EMT, CSCs, and cancer aggressiveness [62], we found that ectopic expression of FOXC2 in mouse ovarian cancer cells resulted in the upregulation of genes involved in EMT as well as CSC differentiation and increased tumor growth in mice. In addition, we showed that FOXC2 is involved in vasculogenic mimicry. This is not unexpected considering that FOXC2 is a major determinant of vascular development and remodeling during embryonic development and in adult tissues [2,3,4,5,39,40] and contributes to angiogenesis during cancer progression. For example, in a melanoma xenograft model, mice lacking one copy of Foxc2 exhibited reduced tumor growth, impaired formation of tumor blood vessels, and decreased pericyte coverage [36]. Additionally, FOXC2 expression in esophageal cancer cell lines facilitated vascular tube formation by human umbilical vascular endothelial cells (HUVEC) [87]. Similar observations have been reported in breast and lung cancer cell lines where induction of FOXC2 expression by inorganic phosphate facilitated HUVEC tube formation and migration in the presence of conditioned media from FOXC2-expressing cancer cells [88]. Importantly, HUVEC migration was completely abolished in the presence of conditioned media from cancer cells in which FOXC2 was knocked down, demonstrating that FOXC2 is a crucial factor in cancer cell-mediated angiogenesis [88]. Mechanistically, FOXC2 was found to be necessary for the expression of SPP1 in cancer cells (path A in Figure 7). Exogenous or tumor-secreted SPP1 has been shown to directly act on endothelial cells to promote angiogenesis via AKT activation [88,89,90]. Consistent with these data, we found that ectopic expression of FOXC2 in mouse ovarian cancer cell lines resulted in Spp1 upregulation and Akt activation. Angpt2, which we found to be upregulated in FOXC2-expressing mouse ovarian cancer cell lines, has been previously identified as a mediator of tumor angiogenesis [72,73,91]. FOXC2 has been shown to control Angpt2 expression by direct activation of its promoter in adipocytes from transgenic mice that overexpress FOXC2 [91]. Studies in hepatocellular carcinoma also showed that transcriptional expression of the Angpt2 promoter was directly targeted by FOXC2 and that Angpt2 knockdown abolished FOXC2-facilitated angiogenesis and tumor growth, suggesting that FOXC2 promotes tumor progression via regulation of Angpt2 [30].

In addition to the well-documented paracrine effects of FOXC2 on endothelial cells, it has been shown that FOXC2-expressing cancer cells are capable of trans-differentiation into endothelial cells. Induction of EMT in a weakly tumorigenic MCF-7 breast cancer cell line promoted trans-differentiation of cancer cells into CD31-positive endothelial cells, which facilitated vascularization and growth of tumor xenografts in mice [92]. Mechanistically, FOXC2 was shown to be a key factor in the acquisition of endothelial phenotypic and functional characteristics in vitro and in vivo (path B in Figure 7) [92].

We did not observe upregulation of prototypical endothelial cell markers (CD31, CD34, VE-cadherin, Icam1, and Vcam1) upon ectopic expression of FOXC2 in mouse ovarian cancer cells. Rather, we observed upregulation of mural cell markers, including Pdgfrb and Cspg4, which have been identified as drivers of VM in breast cancer, glioblastoma, and melanoma through trans-differentiation of CSC-like cells to mural cells [78,79,80]. Our analysis of FOXC2 overexpression in mouse ovarian cancer cell lines suggests that, in addition to promoting EMT and CSC differentiation, FOXC2 might be involved in VM (path C in Figure 7). We confirmed that FOXC2 can promote VM in human ovarian cancer cell lines in vitro and showed that vascular channels indicative of VM are present in FOXC2-expressing mouse and human ovarian cancers. Of note, we observed VM in a smaller percentage of HGSOC than previously reported (10% in our study vs. 25–37% in previous studies [47,48,49,50,51]). This is probably because we used a revised criterion to define VM, which requires the presence of red blood cells within a lumen enclosed by epithelial PAS-positive/CD31-negative epithelial cells [55].

Although ovarian cancers were among the first cancer types in which VM was identified and associated with poor prognosis [47,48,49,50,51], little is known about the molecular mechanisms that support VM in ovarian cancer. Recently, the PAX2 transcription factor, which is upregulated in a subset of HGSOC, was shown to induce VM in vitro and in vivo [60]. Our study contributes to the understanding of VM in ovarian cancer by identifying FOXC2 as another transcription factor involved in VM in ovarian cancer.

In our study, only 12% of HGSOC were positive for FOXC2 and the expression was restricted to a small subset of cancer cells. In a recent immunohistochemistry analysis of ovarian tumors, FOXC2 expression was detected in more than 50% of HGSOC [93]. The polyclonal FOXC2 antibody used in that study was primarily expressed in cancer cells although the nuclei of stromal cells also exhibited weak expression [93]. We suspect that a higher specificity of our monoclonal FOXC2 antibody obtained from Dr. Naoyuki Miura explains why we observed a smaller proportion of FOXC2-positive HGSOC. Additionally, given the focality of FOXC2 expression that we observed in our cases, it is possible but unlikely that we missed some positive tumors among the 30 specimens that were analyzed as TMA cores. It is unlikely because we also identified only three positive HGSOC samples among 20 samples that were stained as whole-slide sections. The major limitation of our study is the small number of HGSOC samples and limited clinical data, which preclude correlative analyses of FOXC2 expression and the presence of VM with patient survival. Both FOXC2 expression and the presence of VM have been shown to correlate with poor survival in previous ovarian cancer studies [47,48,49,50,51,93].

## 5. Conclusions

Our study confirms the previously recognized roles of FOXC2 in cancer and introduces VM as one of the processes by which FOXC2 could promote cancer progression. Although our study was focused on ovarian cancer, FOXC2 has been shown to induce EMT and CSC differentiation in other aggressive malignancies [85], and EMT-facilitated CSC differentiation has been linked to VM in several cancer types [77,94]. Thus, it is likely that FOXC2 is involved in VM in cancers other than HGSOC.

## Data Availability

The raw and processed data presented in this study are available upon request from the corresponding author.

## Supplementary Materials

The following supporting information can be downloaded at: https://www.mdpi.com/article/10.3390/cancers14194851/s1, Figure S1: Wound-healing scratch assay; Figure S2: The proliferation of SKOV3 cells transduced with an empty vector (EV) or FOXC2 was measured by luminescence using the CellTiterGlo kit; Figure S3: Comparative measurement of parameters obtained from the vascular mimicry assay image analyses of SKOV3 and TYKNU cell lines in which FOXC2 was overexpressed and silenced, respectively; Table S1: RNA-seq analysis of 20 genetically defined mouse ovarian cancer cell lines with ectopic expression of FOXC2 or GFP. The values represent normalized read counts. File S1. Full pictures of the Western blots in Figure 1D.
