# Supplementary material for: FOXC2 Promotes Vasculogenic Mimicry in Ovarian Cancer
Source: Cancers (Basel). 2022 Oct 4;14(19):4851. doi: 10.3390/cancers14194851 (PMC9564305; doi:10.3390/cancers14194851)
Supplement: Supplementary file 1 [file cancers-14-04851-s001.zip › FIGURES Suppl Revised.pdf]

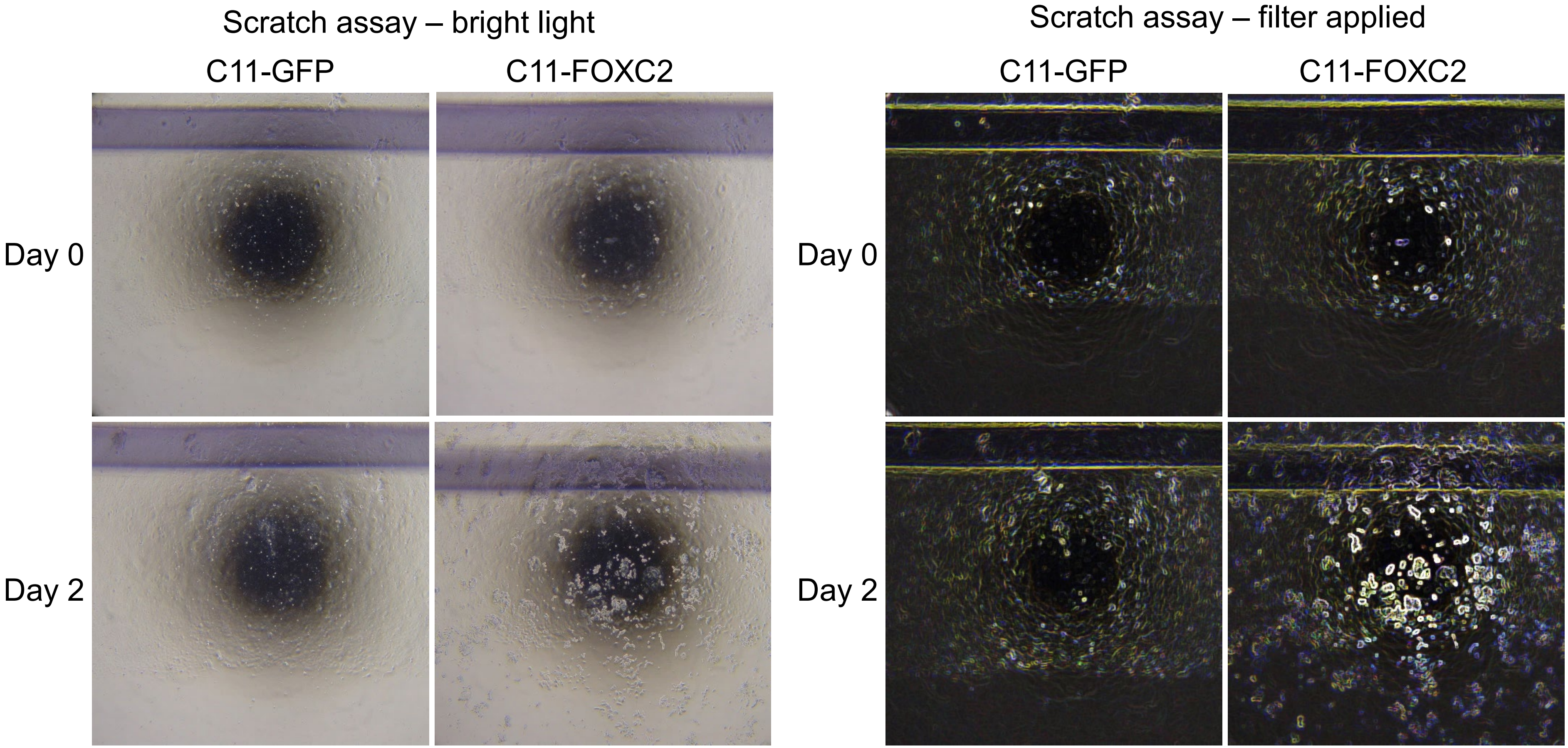

Figure S1. Wound-healing scratch assay. 4×10^6 cells were plated in triplicate in a 6-well plate containing 10 µg/ml mitomycin C to block proliferation. Upon reaching confluence, cells were scratched using a 200 µl pipette and the medium was replaced. The images show the same area of the plate on Day 0 and Day 2 after scratching the plate. A dark filter was applied to the images on the right to visualize the wound border. On Day 2, C11-FOXC2 cells appeared to detach from the monolayer and float in the media.

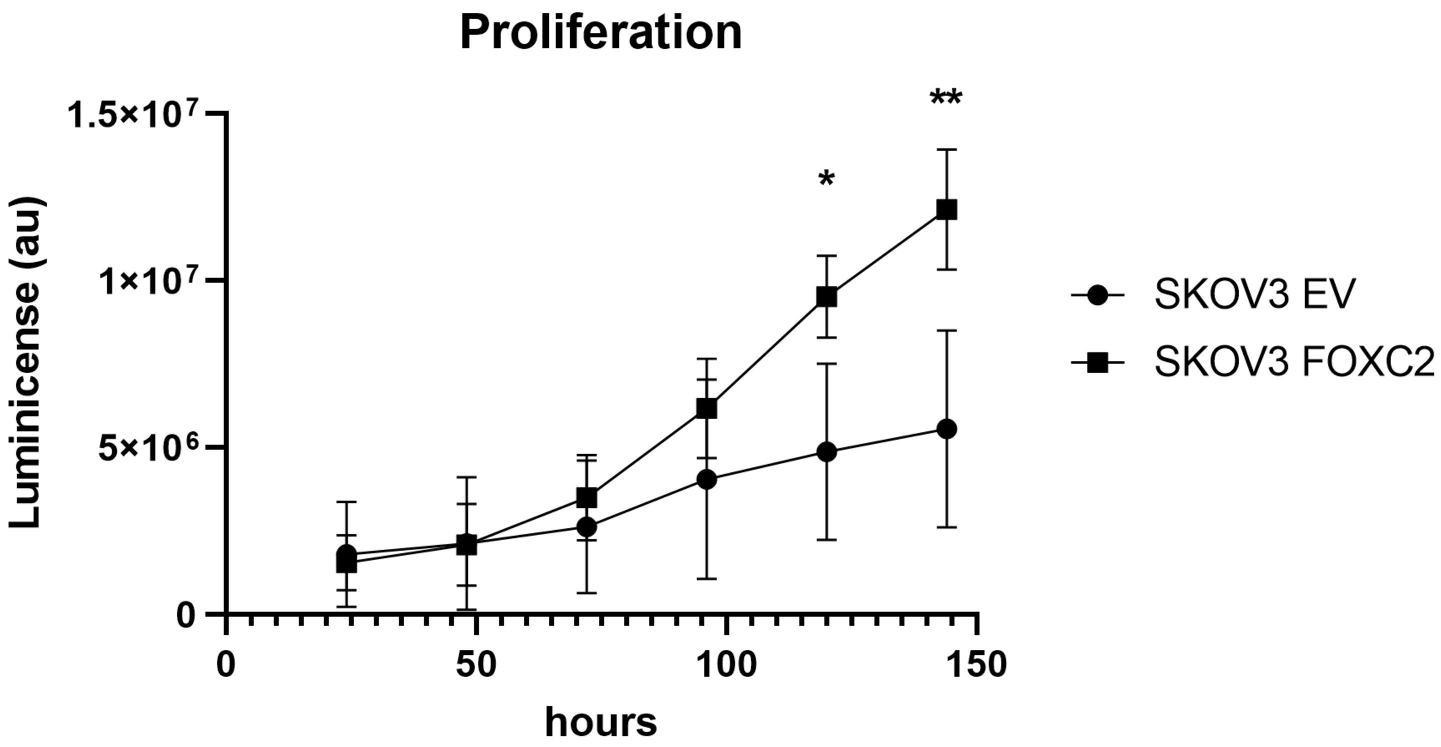

Figure S2. The proliferation of SKOV3 cells transduced with an empty vector (EV) or FOXC2 was measured by luminescence using the CellTiterGlo kit.

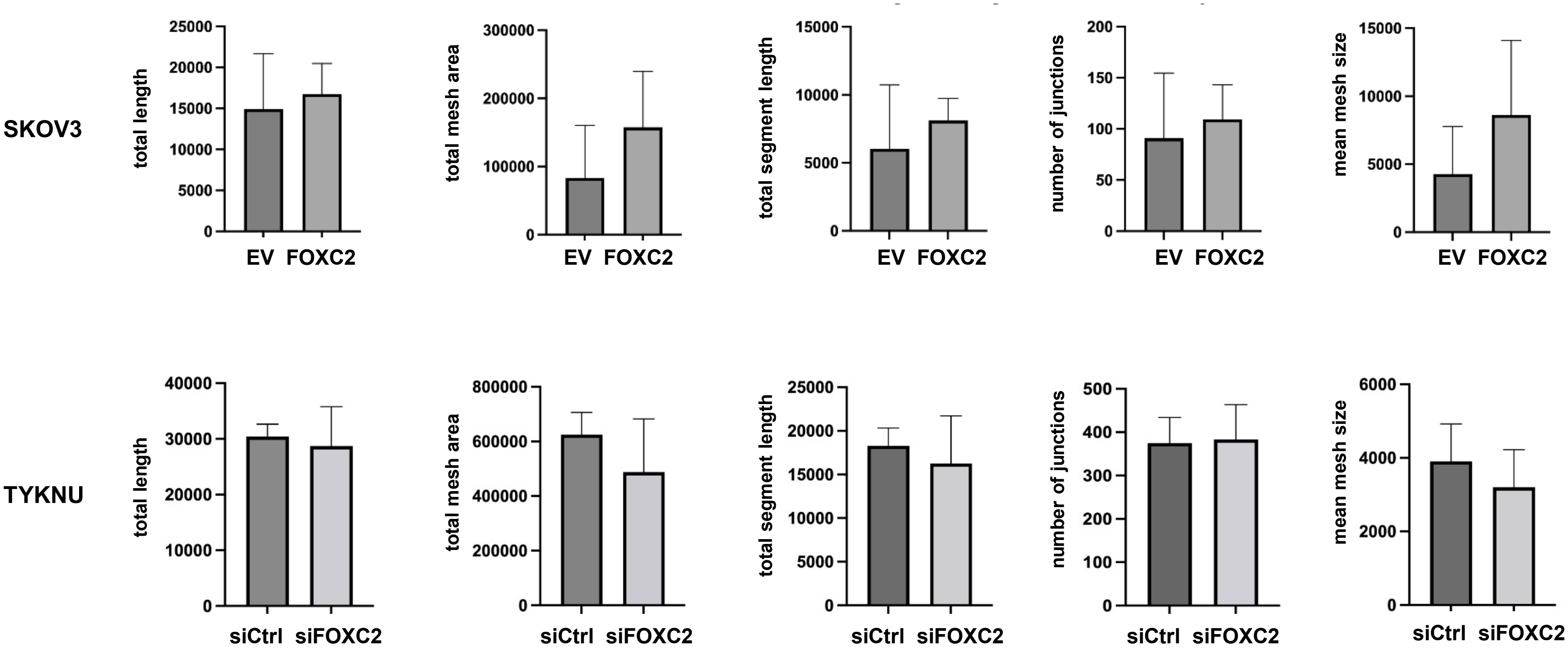

Figure S3. Comparative measurement of parameters obtained from the vascular mimicry assay image analyses of SKOV3 and TYKNU cell lines in which FOXC2 was overexpressed and silenced, respectively. Microphotographs were taken every 3 hours by the MuviCyte live cell imaging software and analyzed in Image J with an angiogenesis plugin. Error bars represent SEM of technical triplicates. EV, empty vector; siCtrl, short interfering RNA control.
