# Supplementary material for: FOXC2 Promotes Vasculogenic Mimicry in Ovarian Cancer
Source: Cancers (Basel). 2022 Oct 4;14(19):4851. doi: 10.3390/cancers14194851 (PMC9564305; doi:10.3390/cancers14194851)

Figure 1D Western Blots

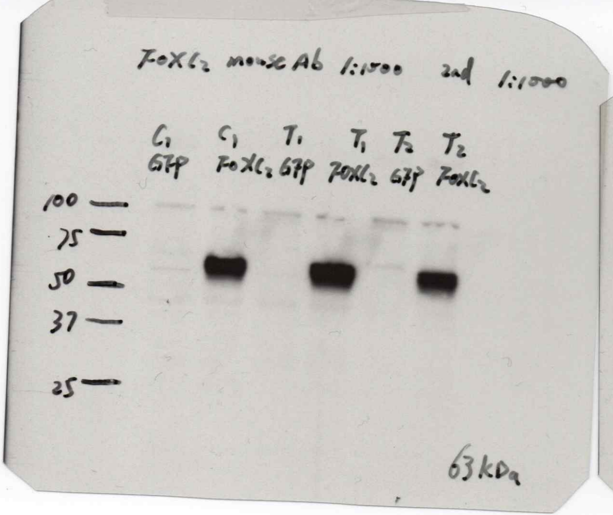

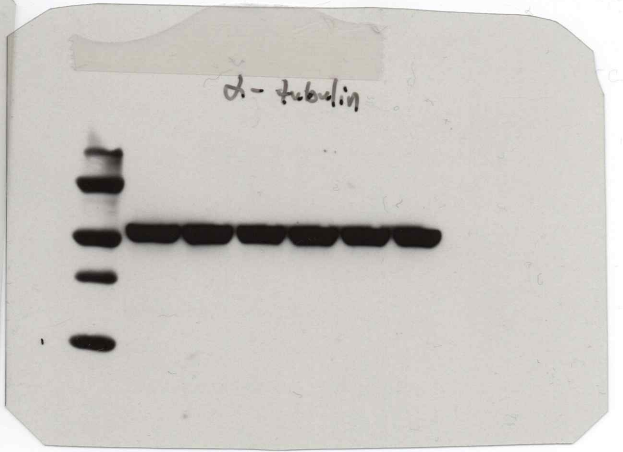

Supplement: Supplementary file 1 [file cancers-14-04851-s001.zip › File S1. Western Blots full.pdf]
